# Emerging Technologies in Community-Based Older Adult Care: The Perception of Nurses

**DOI:** 10.1097/jnr.0000000000000687

**Published:** 2025-06-24

**Authors:** Sivia Barnoy, Tal Soffer, Yoel Raban, Irit Schwartz-Attias, Mali Kusha, Hagar Wechsler, Sigalit Warshawski

**Affiliations:** 1 Department of Nursing Sciences, School of Health Professions, Gray Faculty of Medical and Health Sciences, Tel-Aviv University, Israel; 2 Center for Technology and Society Foresight, School of Education, Tel Aviv University, Israel; 3 Meir Academic Nursing School, Meir Medical Centre, Clalit Health Services, Israel; 4 Assisted Living and Geriatric Medical Center Neve Amit, Meuhedet Health Service, Israel; 5 Nursing Division, Meuhedet Health Service, Israel

**Keywords:** emerging technologies, nurses, community nursing, care of older adults

## Abstract

**Background:**

Concurrent with a growing shortage of nurses, the number of older adults living with chronic conditions and disabilities in the community is increasing. Emerging technologies present solutions that may impact nurses’ training and patient care.

**Purpose:**

This study was designed to explore (a) the familiarity of nurses with emerging technologies, their need for additional training, and their perception of the effects of these technologies on the care of older adults in the community and (b) the relationships between the research variables and, respectively, sociodemographic and professional data.

**Methods:**

A descriptive, cross-sectional design was used, and a structured questionnaire was distributed to community nurses throughout Israel between November 2022 and February 2023.

**Results:**

Positive associations were found between nurses’ familiarity with technology, the need for additional training, and the perceived general and specific effects of these technologies on older adult care. Moreover, technological familiarity and the need for training predicted the perceived impact of these technologies on older adult care.

**Conclusions/Implications for Practice:**

The results highlight the importance of familiarizing nurses in community settings with emerging and advanced technologies. This may promote and improve the quality of care provided to older adults in the community. Moreover, continuing training and education for nurses on emerging technologies are essential to familiarize nurses with new technologies, increase technology acceptance, and enhance the quality of care provided to older adults in the community.

## Introduction

The world’s population is aging at an increasing rate. The outlook for the next decades indicates a future increase in life expectancy concurrent with a continued significant rise in older adult populations (65 years and older) in all developed countries. The proportion of older adults in the world’s population is expected to reach 16% by 2050 (United Nations Department of Economic and Social Affairs, Population Division 2021; World Health Organization, 2021).

Researchers and policymakers point to community-based aging as a potential solution that will ensure the provision of essential assistance to older adults and the opportunity for them to live in better conditions in line with their wishes and to enjoy a good quality of life (Bouchard & Gaboury, 2017). The issue of “aging in the community” is receiving considerable attention in the research literature (Chen et al., 2023; Woolrych et al., 2022). This issue integrates multiple aspects, including housing and environment, community, social services, assistive devices and technology, health care, independent functioning, and more (Bigonnesse & Chaudhury, 2020; Schomakers & Ziefle, 2023).

Nurses, being vital to inpatient and community health services, comprise the largest professional group within the health care sector. Community nursing has been widely recognized in health promotion, disease prevention, and disease management for different population groups, including older adults with multimorbidity (Xu et al., 2022). Nurses also play a central role in introducing, implementing, and using digital and technological solutions in health care (Conte et al., 2023).

In Israel, almost one-third of all nurses are employed in community health services (Israeli Ministry of Health, 2022). Most community nurses are employed by one of the four health care organizations responsible for organizing and providing care in the community within the framework of the national health insurance law. However, changes in the health system, patient, and caregiver needs, as well as technological innovations, require nurses to change their work environment and traditional roles. The nursing profession must adapt to meet the needs of worldwide “aging in the community” trends. One example of related changes includes integrating pharmaceutical/therapeutic measures with advanced technological equipment. Advanced technologies are essential to improving care, and emerging technologies may significantly impact the quality of life and care experienced by older populations (Schomakers & Ziefle, 2023; Wong et al., 2022).

In the last decade, significant efforts have been made to deal with changes to the nursing role necessitated by health care becoming more accessible and more available in the community, and focusing more on personal well-being and including more areas of responsibility as well as dynamic and long-term interactions (Irani et al., 2021). At the forefront of these efforts are emerging technologies that aim to harness increasingly sophisticated technological capabilities for the benefit of changing clinical practices, with an emphasis on improving and strengthening care and support for older adults living in the community (Phillips et al., 2020). The most prominent avenues of progress include the application of information technologies, remote medicine, smart homes, artificial intelligence systems, the Internet of Things (IoT), and big data (Huter et al., 2020; Qian et al., 2021).

Despite the rapid uptake and evolution of technological innovations in health care and the professional changes in the nursing profession, the evidence in the literature indicates a discrepancy between the perceptions and adoption of emerging technologies among nurses. The literature review conducted for this study primarily uncovered findings regarding the use of health information technologies among nurses and the paucity of information related to emerging technologies. Current research findings indicate that the general experiences and perceptions of nurses toward using various types of technology (electronic health records, large databases, crowdsourcing, and biosurveillance) are positive (Gaughan et al., 2022; Konttila et al., 2019). However, nurses also indicate related challenges to usage, including inadequate training and support, lack of interoperability, risk of reduced quality of care, increased costs, and higher risk of errors (Gaughan et al., 2022). Nurses perceive developing digital skills and navigating and becoming familiar with new technology to be overly time-consuming and adding to their workload (Brown et al., 2020). This finding is consistent with other studies that have shown implementing new technologies in health care demands more time from hospital staff (Arguilera et al., 2020) and community nurses (Lezard & Deave, 2021). Additional barriers described in the literature that may affect the willingness and motivation of health care professionals to use technology include personal attitudes and experiences, individuals’ competencies, and organizational factors (Conte et al., 2023; Konttila et al., 2019).

Nurse familiarity with emerging technologies, training needs, and attitudes toward the effects of using these technologies in the care of older adults have been underexplored in the literature as main research variables. Familiarity with technology is defined as “a specific activity-based knowledge built on previous experience or learning of how to use a particular interface” (Gefen, 2000, p. 727). Studies conducted on the public and health care professionals to explore the associations between familiarity with emerging technologies and their adoption point to a positive association between these two variables (Chandio et al., 2024; Gefen, 2000; Horowitz et al., 2023). Participants with familiarity and expertise with a technology who understand it and its functions may recognize its possibilities and limits, leading to more positive attitudes toward the technology and its future adoption (Hartwick & Barki, 1994).

The perceived need of nurses for additional training in the use of advanced technologies has been discussed in the literature as a barrier to acceptance (Brown et al., 2020; Gaughan et al., 2022; Konttila et al., 2019) and as a strategy for enhancing technology acceptance (Brown et al., 2020; Iyanna et al., 2022). In this context, training refers to acquiring skills (operating and critical thinking) and knowledge for using advanced technologies (Conte et al., 2023). Among practicing nurses, the literature emphasizes the importance of continuing education and training in promoting positive attitudes toward new technology adoption as well as enhancing care efficiency and patient outcomes (Brown et al., 2020; Conte et al., 2023; Gaughan et al., 2022). While the evidence is currently unclear regarding the percentage of nurses who report lack of knowledge and skills as barriers to operating digital and advanced technologies, most nurses report being willing to receive additional training and experience to facilitate the use of these technologies in health care delivery (Brown et al., 2020; Conte et al., 2023; Gaughan et al., 2022; Konttila et al., 2019).

Reviews of the literature on the perceptions of nurses toward the effect of emerging technologies on older adult care report that only a few studies on this topic have been conducted. Kang et al. (2023) explored South Korean nurses’ perceptions regarding care robots for older adults. The results indicate the potential benefits of using care robots in the care of older adults with dementia or living alone that include promoting noncontact care during the COVID-19 pandemic and promoting older adults’ well-being (reminding them to take medicine and playing games, which may improve cognition and socialization). Similar findings were reported by Rantanen et al. (2018) among Finnish home care nurses regarding the introduction and use of care robots in home care. Respondents expressed perceiving that care robots could be useful in promoting the safety of older people living at home through providing reminders and guidance necessary to maintain prescribed health care schedules. Similarly, McNamara (2024) found that nurses viewed telemonitoring positively when they perceived it as an effective or important tool for preventing falls and promoting patient safety.

In this study, an integrated conceptual framework based on the Mere Exposure Effect (Zajonc, 1968) and the Theory of Planned Behavior (Ajzen, 1985) was used. Combining these models provides a comprehensive framework for understanding how attitudes toward the effect of technologies on older adult care are associated with technology familiarity and the need for additional training among community nurses.

The Mere Exposure Effect (Zajonc, 1968) suggests that individuals develop a preference for things largely due to familiarity. Repeated exposure to a stimulus increases familiarity, which in turn increases positive attitudes toward the stimulus. This model implies that repeated exposure to certain ideas, practices, or environments leads to more positive related attitudes. For instance, employees who are frequently exposed to a new technology in a supportive training environment may develop a more favorable attitude toward using that technology.

The Theory of Planned Behavior (Ajzen, 1985) posits that individual attitudes toward a behavior, subjective norms, and perceived behavioral control shape related intentions and, ultimately, behavior. Knowledge and training are crucial components that influence attitudes and perceived control. According to this theory, attitude is the degree to which a person has a favorable or unfavorable evaluation of a behavior. Knowledge about the outcomes, benefits, and costs of a behavior is essential in shaping this attitude. Training that provides detailed information and clarifies the implications of a behavior enhances this component. Training programs that provide comprehensive information about a behavior can change related attitudes by increasing awareness of the benefits and reducing misconceptions or fears. For example, educating nurses about the advantages of a new technology can positively influence their attitudes toward adoption.

Based on the above and the aforementioned gap in the literature, this study was designed to explore (a) the familiarity of nurses with emerging technologies, their need for additional training, and their perception of the effects of these technologies on the care of older adults in the community and (b) the relationships between the research variables and, respectively, sociodemographic and professional data.

## Methods

### Design and Setting

A descriptive, cross-sectional design was used. The study survey was distributed via an electronic link on the QUALTRICS cloud platform to nurses across Israel working at primary-community clinics under two large health care organizations providing community health services. Data collection was conducted between November 2022 and February 2023. The minimum sample size required to achieve a power of 0.80 and a Cronbach alpha value of .05 was 109 (calculated using the WINPEPI COMPARE2 program).

### Measures

A structured questionnaire in Hebrew was developed based on the findings of an expert survey conducted on 76 experts in the field of medical technologies and geriatrics in Israel and Europe (Soffer et al., 2024). This preliminary work utilized horizon scanning and an expert survey that leveraged the Delphi method to map emerging older-adult-care–related technologies and their potential for use in community health care settings. The experts identified 14 emerging technologies (e.g., autonomous assistive robots, wearable technology) and their materialization time. On the basis of this list, the research team developed the survey tool, which was subsequently pretested on 15 community-based volunteer nurses. In addition, three nursing content experts reviewed the tool for content validity and clarity. Based on the results, several grammatical amendments were made.

The final tool included the following four sections:

1. Sociodemographic and professional data: age, gender, country of birth, religion and religiosity, professional and academic education, place of work, clinical area, length of professional experience, job title, and post-basic courses.
2. Familiarity with emerging technologies in older adult community care. Respondent familiarity with each of the 14 expert-identified emerging technologies was scored on a Likert scale ranging from 0 (*not familia*r) to 5 (*very familiar*). Total possible scores for this section range from 0 to 70, with higher scores indicating a higher level of familiarity with all 14 technologies. The Cronbach’s alpha was .91.
3. Perceived need for training in emerging technologies for older adult care. This section covers the same 14 items described in section B, with respondents asked to rate the degree to which they feel the need for training in each. Answers were given on a Likert scale ranging from 0 (*not at all*) to 5 (*very much*). Total possible scores for this section range from 0 to 70, with higher scores indicating a greater need for training on all 14 technologies. The Cronbach’s alpha was .98.
4. Perceived effects of emerging technologies on older adult care. This section covers the same 14 items described in section B, with respondents asked to rate the perceived general impact of each on older adult care as well as on five areas of need, namely: mobility, cognition, self-care, social life, and accessibility of health services. Each item was ranked on a 6-point Likert scale from 0 (*not at all*) to 5 (*very much*). To assess the impact of the technologies in general as well as in each area, the items were summed, with higher total scores indicating a more significant general effect of these technologies on the care of older adults in the community and on the five areas of need. The Cronbach alpha was .96 for general effect, .92 for mobility, .91 for cognition, .92 for self-care, .91 for social life, and .92 for accessibility of health services.

### Procedure

After receiving approval from the Helsinki committee in the two health care organizations and from the Tel Aviv University ethics committee, an electronic link was distributed to all community nurses with responsibilities for caring for older adults via the organizations’ listserv. To ensure data credibility, the electronic link was distributed only to nurses registered as currently employed in community settings by the two health care organizations. All of the participants signed informed consent prior to completing the survey tool.

### Data Analysis

Pearson coefficients were used to examine associations between the research variables and the targeted sociodemographic and professional variables. *t* tests for independent samples were performed to examine the differences in the research variables by gender (female, male), cultural group (Israeli-Jewish, Israeli-non-Jewish), and religiosity (secular, traditional-religious). A multiple regression analysis was performed to examine whether familiarity with the technologies and training needs predicted participant perceptions of the impact of the technologies on older adult care. The two independent variables were familiarity with the technologies and the need for training, and the one dependent variable was the impact of the technologies on community-dwelling older adult care.

### Ethical Considerations

The study received approval from the Tel Aviv University ethics committee (#005472-1). In addition, the study received approval from the Helsinki committees of two health maintenance organizations (HMOs) responsible for providing community-based health care through clinics across Israel. One approved the research and provided an exemption from Helsinki Committee approval, while the other approved the research (#05-31-08-22).

## Results

The sample consisted of 206 registered nurses from various community health clinics nationwide under the two HMOs. The mean age of participants was 44.32 (*SD*=11.11) years, and the mean years of professional experience was 18.12 (*SD*=11.84). Most were women (92.72%), Jewish-Israeli (78.16%), and Israeli-born (65.53%). About half held a bachelor’s degree in nursing (45.63%) and were working at a primary health clinic in the community (61.65%) as a staff nurse (72.33%). The sociodemographic and professional details for the sample are summarized in Table 1.

**Table 1:** Participant Sociodemographic and Professional Variables (*N*=206)

Variable	*M*	*SD*
Age (year)	44.32	11.11
Length of experience (year)	18.12	11.84
	*n*	*%*
Gender		
Male	15	7.28
Female	191	92.72
Place of birth		
Israel	135	65.53
Other	71	34.47
Religion		
Jewish	161	78.16
Muslim	31	15.05
Other	14	6.79
Education		
Bachelor	94	45.63
Master	90	43.69
PhD	3	1.46
Other	19	9.22
Workplace		
Primary clinic	127	61.65
Specialized clinics	20	9.70
Other	36	17.48
Community	21	10.19
Mental health	2	0.97
Job role		
Management	55	26.70
Nurse	149	72.33
Other	2	0.97
Post-basic course in nursing		
None	37	17.96
Primary health care	35	16.99
Clinical instructor	31	15.05
Intensive care unit	26	12.62
Public health	24	11.65
Other	53	25.72

### Familiarity With Emerging Technologies Related to Older Adult Care

Mean scores and standard deviations for familiarity with each technology are presented in Table 2. The technologies with the highest familiarity scores were telehealth (2.97±1.62), portable diagnostics (2.54±1.59), and voice-activated devices (2.34±1.69), while those with the lowest familiarity scores were exoskeletons (0.94±1.35), autonomous assistive robots (0.96±1.36), and the Internet of Things (1.06±1.48).

**Table 2:** Familiarity, Need for Training, and Perceived Effects of Emerging Technologies on the Care of Older Adults (N = 206)

Technology	*M (SD)*		
	Familiarity	Need for Training	Perceived Effects on the Care of the Elderly
Telehealth	2.97 (1.62)	3.14 (1.70)	3.85 (1.39)
Wearable technology	1.40 (1.60)	2.61 (2.11)	3.44 (1.55)
Portable diagnostic	2.54 (1.59)	3.03 (1.84)	3.61 (1.40)
Health and emotion monitoring	1.44 (1.60)	2.79 (2.02)	3.63 (1.45)
Fall prevention devices	1.22 (1.56)	2.74 (2.07)	4.11 (1.44)
New drug release mechanisms	1.65 (1.62)	2.66 (2.00)	3.88 (1.34)
Smart medication dispensing	1.47 (1.66)	2.78 (2.10)	4.00 (1.37)
Exoskeletons	0.94 (1.35)	2.72 (2.09)	2.99 (1.71)
Internet of Things	1.06 (1.48)	2.61 (2.07)	3.20 (1.55)
Virtual, augmented, and mixed reality	1.32 (1.60)	2.67 (2.10)	3.13 (1.70)
AI-enabled health smart apps	1.67 (1.66)	2.76 (2.09)	3.50 (1.48)
Voice-activated devices	2.34 (1.69)	2.81 (1.94)	3.58 (1.58)
Wound care technology	2.14 (1.78)	3.03 (1.89)	3.94 (1.36)
Autonomous assistive robots	0.96 (1.36)	2.47 (2.18)	3.32 (1.50)

### Perceived Need for Training in Emerging Technologies Related to Older Adult Care

Mean scores and standard deviations for perceived need for additional training in the usage of emerging technologies are also presented in Table 2. The technologies with the highest perceived training needs were telehealth (3.14±1.70), wound care technology (3.03±1.89), and portable diagnostics (3.03±1.84), while those with the lowest perceived training needs were autonomous assistive robots (2.47±2.18), wearable technologies (2.61±2.11), and the Internet of Things (2.61±2.07).

### Perceived Effect of Emerging Technologies on Older Adult Care

Mean scores and *SD*s for the general perceived effect of emerging technologies on older adult care are also presented in Table 2. the participants reported a generally high impact on older adult care, the technologies identified as having the highest perceived effect were fall prevention devices (4.11±1.44), smart medication dispensing (4.0±1.37), and wound care technology (3.94±1.36). Exoskeletons (2.99±1.71), virtual, augmented, and mixed reality (3.13±1.7), and the Internet of Things (3.2±1.55) were identified as having the lowest effect on older adult care.

### Associations Among the Main Research Variables

Significant positive correlations among technology familiarity, need for additional training (*r*=0.22, *p*<.01), general effect on older adult care (*r*=0.28, *p*<.01), and the areas of mobility (*r*=0.33, *p*<.01), cognition (*r*=0.34, *p*<0.01), self-care (*r*=0.26, *p*<.01), and access to health services (*r*=0.35, *p*<0.01) are shown in Table 3. Specifically, nurses with greater perceived familiarity with a technology reported a greater need for related training and a higher impact of that technology on older adult care, both in general and in the areas of mobility, cognitive function, self-care, and access to health services. Moreover, positive and moderate correlations were found between need for training and the general effect on older adult care (*r*=0.38, *p*<.01), mobility (*r*=0.26, *p*<.05), cognitive care (*r*=0.27, *p*<.05), and self-care (*r*=0.35, *p*<.01). These results indicate that greater perceived need for training in an emerging technology correlates with the perception that this technology may affect older adult care in general as well as in the areas of mobility, cognition, and self-care.

**Table 3:** Correlation Matrix Among Research Variables (*N*=206)

	Variable	*M (SD)*	Range	1	2	3	4	5	6	7	8
1.	Familiarity	22.81 (14.7)	0–5	1							
2.	Need for training	36.06 (26.05)	0–5	.22^*^	1						
3.	General effect	44.27 (21.45)	0–5	.28^*^	.38^*^	1					
4.	Mobility	43.45 (20.05)	0–5	.33^*^	.26	.47^*^	1				
5.	Cognition	43.62 (19.24)	0–5	.34^*^	.27	.43^*^	.93^*^	1			
6.	Self-care	49.41 (19.26)	0–5	.26	.35^*^	.55^*^	.80^*^	.80^*^	1		
7.	Social life	41.13 (19.71)	0–5	.20	.17	.38^*^	.84^*^	.88^*^	.76^*^	1	
8.	Accessibility of health services	47.06 (18.46)	0–5	.35^*^	.21	.44^*^	.83^*^	.80^*^	.79^*^	.75^*^	1

*p <.01. **p < .05.

### Influence of Sociodemographic and Professional Variables on Main Research Variables

A significant difference was found between men and women in terms of familiarity with the emerging technologies (*t*=3.05, *p* = .003), with men reporting greater familiarity (mean=33.43, *SD*=22.37) than women (mean=21.95, *SD*=13.68). A significant difference between Jewish-Israeli and non–Jewish-Israeli participants was also found in terms of familiarity with the emerging technologies (*t*=2.49, *p* = .01), with Jewish-Israeli participants reporting a lower level of familiarity (mean=21.56, *SD*=14.32) than non–Jewish-Israeli participants (mean=27.76, *SD*=15.53).

### The Relationship Between the Main Research Variables and the Technologies’ Effect on the General Care of Older Adults

A stepwise multiple linear regression was conducted, with the effect of the technologies on older adult care in general treated as the dependent variable and familiarity with the technologies and need for additional training treated as independent variables. No multicollinearity was detected between the independent variables (variance inflation factor [VIF] = 1.053; tolerance = 0.95). The regression model was found to be significant (*F*(2, 119)=14.62, *p*<.001), explaining 20% of the variance in the dependent variable. Familiarity with the technologies and perceived need for training were both found to have unique positive and significant contributions to the perceived effect of technologies on older adult care (*B*=0.30, *SE*=0.11, β=0.23, *t*=2.77, *p*=.007 and *B*=0.26, *SE*=0.06, β=0.33, *t*=3.91, *p*<.001, respectively). Hence, higher levels of familiarity with the technologies and perceived need for training predicted higher levels of perceived effect of the technologies on older adult care in general (Table 4).

**Table 4:** Multiple Linear Regression of the General Effect of Emerging Technologies on Older Adult Care (*N*=206)

Variable	*B*	*SE*	β	*t*	*p*
Familiarity with emerging technologies	0.30	0.11	0.23	2.77	.007
Need for training	0.26	0.06	0.33	3.91	<.001

*Note.* Adjust *R* ^2^= .18, *F*(2, 119)=14.62, *p* <.001.

## Discussion

This study was designed to investigate the familiarity of community nurses with emerging technologies utilized in the care of older adult patients, their self-perceived needs with regard to additional training, and their perceptions regarding the effects of these technologies on older adult care. Information and communication technologies (ICT) such as telehealth, portable diagnostics, and voice-activated devices were found to be the most familiar to nurses. This may be explained by the rapid integration and use of ICT in health care and daily nursing work over the past decade (Huter et al., 2020; Iyanna et al., 2022). Electronic medical records, remote diagnosis and monitoring applications, for treatment and counseling have, especially during the COVID-19 pandemic, become increasingly widespread worldwide (Qian et al., 2021). In terms of self-perceived need for additional training, the nurses identified ICT-related applications (i.e., telehealth, wound care technology, and portable diagnostics) as requiring additional training. This finding echoes a similar perceived need for ICT-application training found in an earlier study on Israeli nurses (Warshawski et al., 2019). Based on this result, nursing faculty and nursing educators should work to introduce and integrate emerging technologies into all courses addressing older adult care in the community. Furthermore, nursing administrators, ideally in the geriatric nursing field, should assess nurse familiarity with emerging technologies and their need for additional training.

In general, the participants perceived using all of the included technologies to positively affect community-based older adult care, with technologies related to or responding to known risks in this population (e.g., fall prevention devices, smart medication dispensing, new drug release mechanisms) perceived most positively. In other words, technologies perceived as useful and valuable to community-based older adult care were perceived as having a stronger positive effect in this care context. This finding corresponds with those of prior research (Allen et al., 2023; McNamara, 2024), indicating that nurses hold positive attitudes toward, for example, telemonitoring when they perceive it as an effective and valuable tool for improving patient safety. These findings emphasize the roles of nurse managers and educators in promoting nurses’ familiarity with a wide range of emerging technologies in the care of older adults, improving their knowledge in this regard, and their acceptance of emerging technologies.

Positive associations were found between familiarity with these technologies, self-perceived need for additional training, and perceptions regarding the potential general and specific positive effects of these technologies on older adult care. A possible explanation may be rooted in the conceptual framework of this study. As described earlier, the Mere Exposure Effect (Zajonc, 1968) suggests that familiarity increases perceived preference. Thus, repeated exposure to a stimulus increases familiarity, which in turn increases positive attitudes toward the stimulus. It may be that familiarity with a technology leads to a better understanding of it and its functions, leading to the recognition that additional training/experience is required to use that technology effectively. In addition, greater understanding of a technology promotes greater awareness of its possibilities and the desire to use. The Theory of Planned Behavior (Ajzen, 1985) may also add to this explanation, as knowledge and training are crucial components that influence attitudes and perceived control. Knowledge about the outcomes, benefits, and costs of behavior is essential in shaping attitudes. Training programs that provide comprehensive information about a behavior can change attitudes by increasing awareness of the benefits and reducing related misconceptions or fears. Support for these explanations can be found in earlier studies on artificial intelligence adoption among health care professionals (Chandio et al., 2024) and the general public (Horowitz et al., 2023). Greater familiarity and expertise with AI and similar technologies were associated with more positive attitudes toward AI use. Direct experience influences how people process information. When individuals self-identify as having experience with an application, they develop greater appreciation for that application, which eventually generates positive attitudes toward the underlying technology and future adoption (Hartwick & Barki, 1994).

Gender and cultural group were found to be associated with differences in the degree of familiarity with emerging technologies, with men and non-Jewish nurses reporting more familiarity than women and Israeli-Jewish nurses, respectively. These findings agree with earlier findings in the literature (Goswami & Dutta, 2015) that indicate men are more likely to use information technologies and e-learning applications than women. Gender has been previously suggested as a factor of influence on technology adoption, as men have been shown to be more technologically adept than women. In terms of cultural groups, an earlier study on Israeli nursing students (Warshawski, 2020) found non–Jewish-Israeli students used significantly more information and communication technologies during clinical placements than their Israeli-Jewish peers. Similar differences in ICT usage were found by Mesch et al. (2012) in the general Israeli population. Israeli Arabs were shown to be more likely than Israeli Jews to search for health information and communicate about health issues online. Moreover, an investigation of differences in social media-based health participation identified Israeli Arabs as more engaged in related activities than their Israeli-Jewish peers (Rosenberg et al., 2021). This may explain the differences in this area found in this study. These findings emphasize the need to promote awareness of emerging technologies among all nurses to facilitate their use in clinical practice. Moreover, nurse educators should consider the influence of technology usage based on sociodemographic characteristics and develop appropriate related support and assistance programs.

The findings also point to the predictive role of familiarity and the need for training in the self-perceived effect among nurses of emerging technologies on older adult care. This finding may be explained through the Theory of Planned Behavior (Ajzen, 1985). A prior study conducted on university students (Taylor & Todd, 1995) demonstrated that perceived behavioral control, including training and familiarity elements, is a significant predictor of attitudes toward technology. Moreover, training enhances perceived control and reduces anxiety, leading to more favorable attitudes.

### Limitations

This study is affected by two main limitations related to the sampling strategy and survey tool. The convenience sampling strategy used may limit the generalizability of the findings to all community nurses in Israel. However, the wide geographical coverage and wide range of community clinics included may have contributed to the variance in the findings. In the future, using a randomized sample is recommended to increase the generalizability of the findings. With regard to the survey tool, it was first used in its current form in Hebrew in this study. Its validity should be re-examined in future studies.

### Conclusions and Recommendations

The findings of this study indicate that nurses employed in community-based older adult care are mainly familiar with ICT that is already part of their daily work in clinics. These technologies are also perceived by nurses as affecting the care of older adults. Nurses feel they need additional training in the use of these technologies, especially those with which they are already familiar, as familiarity is associated with awareness of the need for additional training and of the effect of these technologies on patient care. Thus, it is necessary to promote and develop familiarity with emerging technologies among nurses, especially in clinical practice, to improve care quality and safety. Hence, it is recommended that nurse administrators and educators develop educational workshops for nurses focusing on providing factual information and addressing misconceptions about emerging technologies for older adults in the community, as well as offer practical, hands-on training activities that boost confidence and competence in the use of emerging technologies. Furthermore, these workshops should be integrated into the core nursing student curriculum. To achieve effective training and provide the necessary support, it should be tailored to the participants’ sociodemographic characteristics. In addition, nurse administrators and educators should aim to promote continuous professional development in emerging technologies among practicing nurses as well as integrate education on advanced and emerging technologies into the nursing curriculum. Possible actions include developing and assimilating knowledge update courses for nurses within the workplace, encouraging nurses to participate in conferences dedicated to advanced and emerging technologies in health care, and promoting nurses’ active participation in technology-related committees or quality improvement projects targeting the assimilation of emerging technologies at the organizational level. Regarding nursing education, developing and integrating structured courses and workshops focusing on emerging technologies and their applications in the care of older adults, providing hands-on training, and offering online e-learning platforms hold the potential to expand learning opportunities and promote the familiarity and competencies of nursing students in this area.

The familiarity of nurses with emerging technologies and their need for training contribute positively and significantly to their perceptions regarding the effect of emerging technologies on older adult care. This finding emphasizes the need for continuous professional development with regard to the use of emerging technologies in older adult care, particularly in terms of familiarizing nurses and offering them continuing training with emerging technologies in clinical practice. These may promote and improve the quality of care provided to older adults in the community.
